# Future of Antiretroviral Drugs and Evolution of HIV-1 Drug Resistance

**DOI:** 10.3390/v15020540

**Published:** 2023-02-15

**Authors:** Charlotte Charpentier, Quentin Le Hingrat, Valentine Marie Ferré, Florence Damond, Diane Descamps

**Affiliations:** Service de Virologie, Université Paris Cité, INSERM, IAME, UMR 1137, AP-HP, Hôpital Bichat-Claude Bernard, F-75018 Paris, France

**Keywords:** HIV, drug resistance, antiretroviral, long-acting

## Abstract

Highly active antiretroviral (ARV) therapy has been used for many years, but the use in low- and middle-income countries of antiretroviral drugs with low genetic barrier to resistance, combined with limited availability of viral load testing, has led to higher rates of acquired drug resistance, sustaining the rate of transmitted drug resistance. Here, we describe the evolution of ARV drugs with the ongoing development of injectable long-acting forms and the requirements regarding all new ARV drugs (i.e., no transmitted drug resistance, no cross-resistance and high genetic barrier to resistance). Then, we report the evolution of both transmitted and acquired resistance regarding new ARV drugs. The WHO has set very ambitious but motivating goals for HIV testing, treatment and viral suppression, aiming to achieve rates of 95% for all three by 2025. Reaching these goals requires a wide implementation and use of close virological monitoring in LMICs.

## 1. Future of Antiretroviral Drugs: What to Expect?

### 1.1. Long-Acting Antiretroviral Drugs

Research and development efforts have led to several innovations in the latest years, notably the arrival of new classes of antiretroviral (ARVs) such as capsid inhibitors. However, the most notable axis of development for ARV drugs has concerned their mode of administration, particularly the development of long-acting forms. Thus, the capsid inhibitor lenacapavir (LEN) is administered subcutaneously (sc) every six months. The recent CALIBRATE trial showed the non-inferiority of sc LEN, combined with two oral daily ARV, in ARV-naïve patients [1], and the CAPELLA trial showed a high percentage of virological response of LEN in combination with an optimized background regimen; in patients with virological failure and multidrug-resistant viruses, 86% of them achieved a plasma viral load < 200 c/mL at week 52 [2]. The NNRTI rilpivirine (RPV) and the integrase strand-transfer inhibitor (INSTI) cabotegravir (CAB) have also been developed in a long-acting form. The ATLAS, FLAIR and ATLAS-2M randomized-controlled trials have proven the non-inferiority of the dual-therapy RPV + CAB administered intramuscularly every two months [3,4]. The nucleoside reverse transcriptase translocation inhibitor (NRTTI) islatravir (ISL) is also a long-acting agent. In December 2021, ISL clinical trials have been placed on hold by the FDA due to substantial decreases in lymphocytes and CD4+ T-cell counts at W12 and W24 post initiation. Recently, in September 2022, the FDA authorized the initiation of a phase 2 study evaluating an investigational weekly oral combination treatment regimen of ISL and LEN in people living with HIV (PWH) who are virologically-suppressed, with a lower dose of ISL.

Other models of long-acting forms are under development, including implants; that could enable an annual administration. Currently, there is no phase 3 trial evaluating the long-acting ARV form developed as an implant.

### 1.2. Specific ARV Drug Resistance Characteristics

Several virological objectives must be met by new ARV drugs. Firstly, a high genetic barrier to resistance is expected, as observed with protease inhibitors or 2nd generation INSTI. Secondly, new ARV drugs should ideally select specific and different drug resistance profiles, to be able to be active against mutants selected by first generation ARV in case of virological failure and to avoid cross-resistance within a class of ARV. Interestingly, the most recent NNRTI, doravirine (DOR), presents a specific resistance profile with a low-level of cross-resistance with others NNRTI (first or second generation) [5]. In the phase 3 randomized-controlled clinical trials, virological failure followed by emergence of resistance was very rare, observed in only 1.6% of the patients (n = 12) at year 4 of follow-up [6,7]. Among the seven patients with virological failure and emergence of resistance in the DOR arm, the viruses of four patients remained susceptible to etravirine [6,7]. Thirdly, it is mandatory that ARV susceptibility is not impacted by the presence of polymorphisms present in the viruses of ARV-naïve patients; i.e., the ARV drug is expected not to be impacted by HIV genetic diversity and to be active against all HIV subtypes and Circulating Recombinant Forms (CRF). This will allow the use of this ARV before availability of a pre-therapeutic genotypic resistance test result. The most recent ARV drugs, such as the capsid inhibitor, LEN, the 2nd generation INSTI, DTG and BIC, and the NNRTI, DOR, all fulfilled this criterion.

## 2. Evolution of ARV Drug Resistance

### 2.1. HIV Transmitted Drug Resistance

Regarding the Transmitted Drug Resistance (TDR) in low- and middle-income countries (LMIC), a high and rising prevalence of drug-resistance associated mutations, affecting mainly the NNRTI drug class, was observed, with up to 25% of patients presenting TDR in some countries [8]. These worrying findings, in a context where pre-therapeutic genotypic resistance testing is rarely available, have conducted to a change in the WHO guidelines in 2019 with the introduction of the 2nd generation INSTI, dolutegravir (DTG), as the third agent of the first-line regimen. Despite the very low prevalence of TDR to 2nd generation INSTI [9] and the absence of integrase polymorphisms impacting DTG susceptibility, epidemiological surveys remain crucial. Indeed, virological monitoring of PWH often remains limited, due to restricted access to plasma viral load testing and even more to genotyping, hampering the detection of drug resistance mutations (DRM) in the treated population and the potential transmission of these DRMs to newly infected patients, which would severely and negatively impact HIV infection care.

### 2.2. HIV Acquired Drug Resistance

A recent meta-analysis recorded drug resistance in a high proportion of patients after virological failure on a tenofovir-containing first-line regimen across LMIC region, highlighting that effective surveillance for transmission of drug resistance is crucial [10]. Taking into account the recent wide use of DTG in first-line regimen, even if this drug has a high genetic barrier to resistance, it will be necessary to perform epidemiological resistance surveys regarding acquired drug resistance in LMIC. A recent systematic review and meta-analysis showed the presence of INSTI-resistant variants circulating in Sub-Saharan Africa, even if it concerned a low number of patients [11]. Other studies in LMIC confirmed that the most prevalent DTG resistance-mutations were G118R and R263K [12,13]. These findings showed that viruses harboring INSTI resistance mutations are already circulating in Sub-Saharan Africa.

To this aim, wide implementation of viral load assays is mandatory to diagnose earlier virological failure and to avoid a long duration of virological failure that will lead to the accumulation of drug resistance mutations. Furthermore, lowering the WHO threshold for virological failure currently set at 1000 c/mL could also be discussed. Indeed, a viral replication between 200 and 1000 c/mL allows a risk of drug resistance mutation emergence [14]. Virological monitoring is mandatory to detect virological failures and to reduce the duration of viral replication under ARV selective pressure that can lead to an accumulation of drug resistance-associated mutations. Thus, regarding ADR, what is expected of new drugs is obviously a high genetic barrier to resistance, i.e., limited VF and rare emergence of resistance in case of VF.

Regarding Multi-Drug Resistance (MDR), first there is no consensus regarding the definition of MDR, which can either be defined by the number of drug resistance mutations or the number of ARV that are still active (i.e., therapeutic options left). The number of persons living with highly-mutated viruses is limited, although it is more common for HIV-2 infection. However, when therapeutic options are limited, this can cause large public health issues, especially in LMIC which have a reduced availability of ARV drugs, especially new classes, preventing physicians to switch patients to an efficient ARV regimen.

### 2.3. Resistance Profiles of New ARV Drugs

The use of the recent entry inhibitors (ibalizumab, fostemsavir) is limited to highly-treatment experienced patients with MDR viruses, notably in high-income countries due to their cost. Genotypic resistance determinants to these drugs are not entirely defined and still need to be investigated. Thus, for fostemsavir, the presence of baseline envelope polymorphisms is associated with a reduced phenotypic susceptibility to fostemsavir [15]. However, in the BRIGHTE study, their presence was not linked to virological response at W96 [15]. For IBA, resistance is linked to a decrease in the maximum of percentage Inhibition (MPI) determined by phenotypic analysis and in a loss of N-glycosylation sites in the V5 gp120 loop determined by genotypic analysis [16].

Regarding ISL, there is a reduced phenotypic susceptibility in presence of the M184V mutation. Interestingly ISL remains active in presence of the rare Q151M mutation that confers class-wide resistance to NRTI [17,18].

The most recent NNRTI, DOR, is characterized by a low prevalence of TDR, around 1.4% [19], and exhibits specific resistance mutations pathways, notably the emergence of the F227C mutation, limiting cross-resistance with other NNRTI [6,7]. However, although single NNRTI drug resistance mutations do not lead to an increased phenotypic resistance to DOR, the presence of multiple NNRTI drug resistance mutations can impact DOR activity [20].

The capsid inhibitor LEN has the advantage of presenting neither TDR [21], nor natural polymorphism impacting its phenotypic susceptibility. Drug pressure selection in vitro experiments showed a relatively rapid emergence of resistant viruses and high levels of resistance observed with only a single mutation in the capsid [22]. Importantly, there is no cross-resistance with mutations selected with protease inhibitors [23]. Indeed, protease inhibitors mutations can be accompanied by selection of mutations in the gag cleavage sites. Resistance analyses performed in the randomized controlled phase 3 trials showed the emergence of resistance mutations in case of VF in 2/157 patients in the CALIBRATE trial, including ARV-naïve patients, and in 8/72 in the CAPELLA trial including patients with MDR viruses [1,2]. These data suggest a low genetic barrier to resistance of LEN, and a 2nd generation of capsid inhibitors will probably be needed to improve genetic barrier to resistance.

The INSTI CAB has a weaker genetic barrier to resistance than the 2nd generation INSTI, with a higher fold-change of resistance of the Q148K mutants to CAB than to DTG or BIC [24]. In addition, there is an emergence of drug resistance mutations in the case of VF in CAB-treated patients [3,4], while there was no resistance to INSTI at time of VF in the phase 3 randomized clinical trials assessing the INSTI 2nd generation BIC and DTG.

## 3. Conclusions

The WHO has set very ambitious but motivating goals for HIV testing, treatment and viral suppression, aiming to achieve rates of 95% for all three by 2025, as well as to end new HIV infections in children by 2030. The latest WHO treatment guidelines, with the introduction of the DTG, are an important step forward to achieve virological suppression in all ARV-treated patients. However, despite being a potent ARV and having a high genetic barrier to resistance, the wide use of DTG needs to be cautiously monitored in order to detect potential emergence of resistance mutations which will contribute to resistance transmission. Achieving the WHO goals would be illusory without three mandatory milestones:

- Having access to close virological monitoring, implying a wide availability of viral load platforms and a large use by physicians;
- Lowering the 1000 c/mL threshold of virological suppression to 200 c/mL;
- Increasing the availability of all ARV drugs including BIC and DRV in LMIC.

In conclusion, if we want to overcome the issue of TDR and ADR, the use of ARV drugs with the different criteria we described above is essential. We now have access to such drugs; however, to ensure that their potency remains intact overtime, we must be proactive on the diagnosis of virological failure with a closed virological monitoring in LMIC and with regular epidemiological resistance surveys.

## Data Availability

Not applicable.
